# Comparative Evaluation of Organic and Commercial Treatments Against *Varroa destructor* in *Apis mellifera*: Implications for Honey Yield in Northeastern Mexico

**DOI:** 10.3390/pathogens14101051

**Published:** 2025-10-18

**Authors:** Jesús Humberto Reyna-Fuentes, Oscar Vicente Vazquez-Mendoza, Mirelly Venecia Mireles-Villanueva, Daniel López-Aguirre, Juana Maria Coronado-Blanco, Ruben Alberto Muñoz-Sánchez, Francisco Reyes-Zepeda

**Affiliations:** 1 Facultad de Medicina Veterinaria y Zootecnia, Universidad Autónoma de Tamaulipas, Carretera Victoria-Mante Km 5, Ejido Santa Librada, Ciudad Victoria 87274, Tamaulipas, Mexico; jesushumbertoreyna@gmail.com (J.H.R.-F.); rams14101999@outlook.com (R.A.M.-S.); freyes@docentes.uat.edu.mx (F.R.-Z.); 2 Facultad de Medicina Veterinaria y Zootecnia, Universidad Autónoma del Estado de México, El Cerrillo Piedras Blancas, Toluca 50295, Estado de Mexico, Mexico; osvam2009@gmail.com; 3 Facultad de Ingeniería y Ciencias, Universidad Autónoma de Tamaulipas, Campus del Centro Universitario, Ciudad Victoria 87149, Tamaulipas, Mexico; dlaguirre@docentes.uat.edu.mx (D.L.-A.); jmcoronado@docentes.uat.edu.mx (J.M.C.-B.)

**Keywords:** alternative mite control, organic treatments, sustainable beekeeping, colony health

## Abstract

Infestation by *Varroa destructor* represents one of the major challenges for beekeeping, as it compromises both colony health and honey productivity. The objective of this study was to evaluate the efficacy of different organic treatments for the control of *V. destructor* and their effect on honey production in *Apis mellifera* colonies located on the central region of Tamaulipas, Mexico. A total of 150 colonies were assigned to five treatments: T1, oxalic acid with glycerin; T2, sublimated oxalic acid; T3, Thymol, T4; HappyVarr; and T5, an untreated control. Mite infestation (initial and final) and honey production were analyzed using a non-parametric approach and were evaluated with the Kruskal–Wallis test, and when significant differences were detected, Steel–Dwass multiple comparisons were performed. To examine the relationship between infestation reduction and honey yield, Spearman’s rank correlation was applied. No significant differences were observed in the initial infestation levels. However, final infestation levels showed highly significant differences among treatments (*p* < 0.0001), with T1, T2, T3, and sublimated oxalic acid (T4) significantly reducing mite infestation compared with the control. Sublimated oxalic acid represents the most effective and productive control method under the tested conditions. Honey production also differed significantly among treatments (*p* < 0.0001), with the highest yields recorded in T3 and T4. A strong negative correlation was detected between final infestation and honey production (*p* < 0.0001). In conclusion, treatments based on oxalic acid (particularly sublimated) and HappyVarr proved effective in reducing *V. destructor* infestation and improving honey production, highlighting their relevance as viable alternatives for sanitary management in beekeeping.

## 1. Introduction

The global apicultural industry faces one of its most critical sanitary threats in the form of *Varroa destructor*, an ectoparasitic mite that infests colonies of the Western honey bee, *Apis mellifera*. Originally described on the Asian honey bee (*Apis cerana*), *V. destructor* has since adapted to *A. mellifera*, spreading rapidly across continents and establishing itself as a key driver of colony losses worldwide [1,2]. Its feeding behavior, primarily targeting the fat body tissue and hemolymph of immature and adult bees, results in significant physiological damage, immunosuppression, and reduced life expectancy of host bees [1]. Furthermore, *V. destructor* acts as a vector for a variety of honey bee viruses, including deformed wing virus (DWV), acute bee paralysis virus (ABPV), and Israeli acute paralysis virus (IAPV), thereby amplifying its impact on colony health and survival [2,3].

In regions such as Northeastern Mexico, apiculture plays a pivotal role in both rural livelihoods and agricultural sustainability, contributing significantly to pollination services and honey production. However, the intensification of *Varroa* infestations in recent decades has imposed a severe burden on the economic viability and sustainability of beekeeping operations [4,5]. Chemical acaricides, particularly synthetic miticides such as amitraz, fluvalinate, and coumaphos, have been widely used to manage *Varroa* populations. Nevertheless, the repetitive and indiscriminate application of these compounds has led to the development of mite resistance, contamination of hive products (honey, wax), and adverse effects on bee brood and adult workers [6,7]. These challenges have prompted researchers and beekeepers alike to explore alternative strategies that prioritize bee health, product quality, and environmental safety [8].

Organic treatments defined here as non-synthetic compounds with acaricidal properties have emerged as promising tools in integrated pest management (IPM) programs for *Varroa* control. Substances such as oxalic acid and thymol, derived from natural sources, have shown efficacy in reducing mite loads while maintaining favorable safety profiles for bees and hive products [9,10]. Oxalic acid can be administered through various delivery systems, including sublimation and impregnated glycerin-based towels, allowing flexibility depending on colony conditions and environmental variables [9,11,12]. Thymol, a monoterpenoid phenol extracted from thyme (*Thymus vulgaris*), disrupts mite physiology by interfering with mitochondrial activity and nervous system function [13,14,15]. Recently, some commercial products that combine these or other natural compounds are also available and have been marketed as safe and effective alternatives to traditional synthetic treatments. Despite the growing interest in organic treatments, their field efficacy remains variable and context-dependent, influenced by factors such as colony size, brood presence, environmental conditions, and the method of application [9,11,16]. Most efficacy studies have primarily focused on mite mortality; therefore, incorporating productivity parameters such as honey yield and other hive products is essential for a comprehensive evaluation of the effectiveness and overall impact of the treatments [4,8,11,17]. Given the economic centrality of honey production in regions like Tamaulipas, it is imperative to understand how different *Varroa* control strategies affect both colony health and productive performance under real-world conditions [4,5,17].

In Mexico, the use of natural compounds such as thymol has proven to be an effective alternative for the control of *V*. *destructor*, achieving up to 92.1% efficacy with a 12.5 g dose per application higher than 65% formic acid and comparable to a double thymol dose (88.8%) [18]. Likewise, oxalic acid formulations evaluated in various tropical regions of the country have demonstrated efficacies close to 90%, even under high-temperature conditions and in the presence of capped brood, without evidence of adverse effects or detectable residues in hive products [19]. However, there remains a critical need for region-specific studies to evaluate the practical effectiveness of organic treatments in commercial beekeeping operations, particularly under the semi-arid and subtropical conditions characteristic of Northeastern Mexico. The diversity of management practices among producers, climatic variability, floral resource fluctuations, and the economic pressure to minimize colony losses all demand locally validated *Varroa* control approaches tailored to regional ecological and production contexts [17,20].

The present study aimed to evaluate the acaricidal efficacy of selected organic treatments against *V*. *destructor* and their impact on honey yield in *Apis mellifera* colonies located in six municipalities of Northeastern Mexico. Specifically, we assessed four treatment protocols: (T1) sublimated oxalic acid, (T2) oxalic acid in glycerin-impregnated towels, (T3) thymol, and (T4) HappyVarr^®^ (VEDILab^®^, Queretaro, Mexico) a commercially available product. A fifth group (T5) served as the untreated control. All treatments were applied under field conditions, and honey production was measured at the end of the treatment period to evaluate potential trade-offs between efficacy and productivity. This comparative approach seeks to provide evidence-based recommendations for Mexican beekeepers aiming to implement integrated, residue-free strategies for *Varroa* control while maintaining optimal honey yields.

## 2. Materials and Methods

### 2.1. Study Site

This study was conducted in Ciudad Victoria, Tamaulipas, Mexico, at the Facultad de Medicina Veterinaria y Zootecnia of the Universidad Autónoma de Tamaulipas. This municipality is located in the central region of the state, at approximately 23°44′15″ N and 99°07′59″ W, with a mean altitude of 315 m above sea level. The area lies near the Tropic of Cancer, which contributes to its transitional climatic conditions. The region exhibits a warm sub-humid climate, with a mean annual temperature of 25.5 °C and an average annual precipitation of 533 mm, characterized by a defined rainy season from May to September and a pronounced dry season from November to March [21]. The experimental period was carried out from January to March 2025, corresponding to colony preparation for the upcoming orange tree (*Citrus* spp.) bloom.

### 2.2. Beehive Management

A total of 150 Jumbo-type hives (46.5 cm × 38 cm × 24 cm) were used (30 colonies per treatment), each with an estimated population of 60,000 bees. To monitor natural mite fall, a white cardboard sheet impregnated with commercial shortening based on vegetable oils (corn, peanut, and soybean) was placed on the bottom board of the brood chamber. A mesh screen (4 × 4 mm) was positioned above the sheet to prevent direct contact of the bees with the trap while allowing the counting of fallen *V*. *destructor* mites.

Prior to the trial, colonies were standardized in terms of population and food reserves, consisting of nine brood frames and one internal Boardman feeder (2 L capacity; Boardman^®^, Apiofertas, Aguascalientes, Ags, Mexico). To reduce the potential effects of queen senescence and low oviposition rates, artificially reared queens were introduced, following the methodology described by Doolittle (1889) [22]. The breeding stock originated from genetically pure *Apis mellifera ligustica*, while drones carrying certain Africanization alleles [23] were allowed to freely mate with virgin queens, producing hybrid progeny adapted to the local environment. All queens used in this study were of the same generation and genetic origin.

### 2.3. Application of Treatments

Oxalic acid (OA) (Sigma–Aldrich, St. Louis, MO, USA) was applied to the colonies by crystal vaporization using an electric sublimator (VARROX® vaporizer, Andermatt Group AG, Grossdietwil, Switzerland), specifically designed for the controlled release of the compound within the hive. Prior to each treatment, hives were sealed at the entrance and secondary ventilation openings to prevent vapor leakage and ensure adequate internal saturation. For each colony, 1 g of OA dihydrate crystals was placed in the sublimator chamber, which was inserted through the hive entrance. The device, powered by a 12 V external battery, was operated for 2.5 min, allowing complete sublimation of the compound. Subsequently, the sublimator remained at the hive entrance for an additional 30 s to ensure that the full vapor dose was delivered into the colony. Once the device was removed, hives remained sealed for an additional 10 min to allow homogeneous dispersion of oxalate crystals over the bees and internal hive surfaces, thereby ensuring effective exposure of phoretic mites to the treatment [9]. The procedure was repeated three consecutive times, with 10-day intervals between applications, thereby ensuring continuous exposure of the mite population throughout the experimental cycle.

For the second treatment, colonies were managed using the modified methodology described by Sabahi et al. (2020) [24]. Colonies were treated with the oxalic acid-glycerin towel method, which consisted of impregnating absorbent Scott^®^ shop towels (half a roll, 55 sheets cut in half) with a mixture of oxalic acid dihydrate (505 g) and vegetable glycerin (400 mL; 505 g). The solution was heated to 50–60 °C until complete solubilization of oxalic acid was achieved, ensuring homogeneous absorption of the compound into the towels. Once prepared, the towels were cooled to room temperature and stored in airtight containers until application. Two half towel pieces (equivalent to one full towel) were placed horizontally across the top bars of the brood chamber frames in each hive, allowing bees to move freely over them and thereby maximizing contact with the treatment. The towels remained inside the hives for 10 days, during which oxalic acid was gradually released as bees chewed and walked over the impregnated material. This slow-release mechanism provided continuous exposure of phoretic mites to the acaricide without interrupting brood rearing or queen oviposition. The procedure was repeated three consecutive times, with 10-day intervals between applications.

The thymol (Sigma–Aldrich, St. Louis, MO, USA) treatment was carried out following a modified methodology of [25,26]. For its preparation, 6 g of thymol crystals were dissolved in 6 mL of ethyl alcohol (1:1 dilution), and the mixture was stirred until complete solubilization was achieved. Vermiculite blocks measuring 10 × 5 × 0.5 cm^3^ were then used as absorbent carriers and impregnated with 6 mL of the thymol-alcohol solution, ensuring a homogeneous distribution of the compound throughout the material. Each colony received two impregnated strips, which were placed on top of the brood chamber frames, ensuring their location in high bee-traffic areas to maximize contact. The treatment consisted of three successive applications, each using 6 mL of solution per strip, administered at 10-day intervals.

The HappyVar™ treatment is a commercial product composed of natural oil blends (5 g camphor, 10 g eucalyptus, and 45 g thymol per 100 g). It was applied using two corrugated cardboard pieces (9 × 5 × 0.5 cm^3^), with a dose of 10 mL of the product per hive (5 mL per cardboard), placed at the edges of the brood chamber frames [26]. The procedure was repeated three consecutive times, with 10-day intervals between applications. For the control hives, no treatment was applied, allowing the natural progression of mite infestation.

### 2.4. Honey Production

Honey production in the selected colonies was estimated following the modified methodology described by Guzmán-Novoa and Page (1999) [14]. First, the total number of combs produced by each colony was recorded for each treatment. Subsequently, a random sample of 100 combs from different hives was collected to determine the average weight per comb. The selected combs were uncapped, and their initial weight (combs containing honey) was recorded. They were then placed in a radial extractor to remove the honey. After the extraction process, the combs were removed from the extractor and weighed again to obtain their final weight (empty combs). The amount of honey contained in each comb was calculated as the difference between the initial and final weights. Finally, honey production per colony was estimated by multiplying the total number of harvested combs in each hive by the average honey yield per comb.

### 2.5. Assessment of Varroa *sp.* Control

Treatments were applied on days 1, 10, and 20 after the experiment was initiated. Mite fall on the bottom boards of the colonies was quantified 24 h after each application. To evaluate the effect of the acaricides on the infestation percentages of each colony, the methodology proposed by de Jong (1980) [27] was applied on days 1 and 21 of the trial. In addition, the efficacy of the treatments in reducing mite infestation was determined using the Henderson and Tilton equation [28].

### 2.6. Statistical Analysis

Data on infestation percentage and honey production did not meet the assumptions of normality and homogeneity of variances; therefore, they were analyzed using the non-parametric Kruskal–Wallis test, followed by multiple mean comparisons with the Steel–Dwass method. In this first analysis, results were reported as medians with interquartile ranges. To examine the relationship between infestation reduction and honey yield, Spearman’s rank correlation was applied. In the case of mite fall after treatment applications, data were evaluated using a mixed model repeated-measures analysis of variance, and post hoc comparisons were performed using the Tukey–Kramer test. For both analyses, a probability level of *p* < 0.05 was considered statistically significant. All statistical analyses were conducted using JMP^®^ Pro 17 (SAS Institute Inc., Cary, NC, USA) [29].

## 3. Results

### 3.1. Comparative Efficacy of Acaricides on Final Infestation Levels

The Kruskal–Wallis test revealed highly significant differences in final infestation percentages among treatments (χ^2^ = 76.93; df = 4; *p* < 0.0001). The Steel–Dwass post hoc test showed that all acaricide treatments significantly reduced mite infestation compared with the control group (*p* < 0.0001), whereas no significant differences were detected among the products tested (Table 1).

The oxalic acid sublimated and thymol exhibited the highest efficacy against *V*. *destructor*, with reductions of 77.8% and 77.5%, respectively. Moderate efficacies were observed for oxalic acid/glycerin (72.1%) and the commercial product HappyVarr (72.9%). These results confirm that all treatments effectively reduced mite infestation, with oxalic acid sublimated and thymol showing the most consistent performance (Table 2).

### 3.2. Honey Production

**Figure 1:**
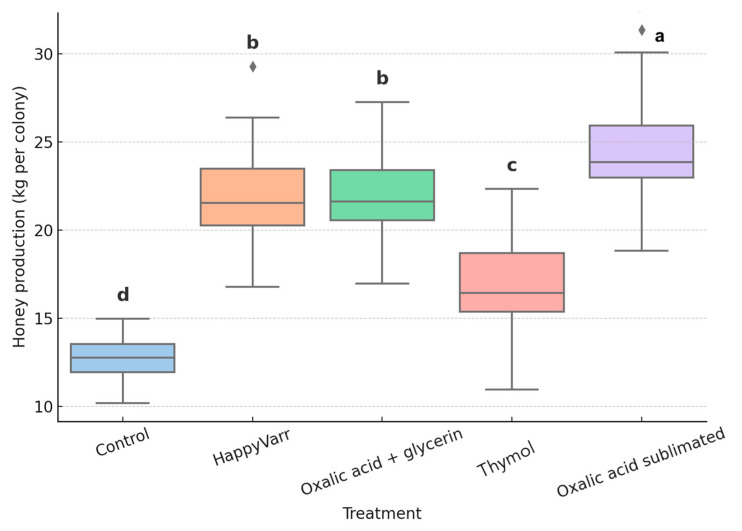
Honey yield (kg per colony) in *Apis mellifera* colonies treated with different organic acaricides. Different letters above the bars indicate statistically significant differences among treatments according to the Steel–Dwass multiple comparison test (*p* < 0.05). Treatments sharing the same letter do not differ significantly.

The Kruskal–Wallis test revealed highly significant differences in honey production among treatments (χ^2^ = 107.51; df = 4; *p* < 0.0001). Colonies treated with oxalic acid sublimated exhibited the highest honey yields (mean = 24.4 kg), significantly surpassing all other groups. Intermediate yields were observed in colonies treated with HappyVarr and oxalic acid + glycerin (both with means = 22.4 kg), which did not differ significantly from each other. Colonies treated with thymol showed moderate honey production (mean = 17.1 kg), while the control group exhibited the lowest values (mean = 13.1 kg) (Figure 1).

**Figure 2:**
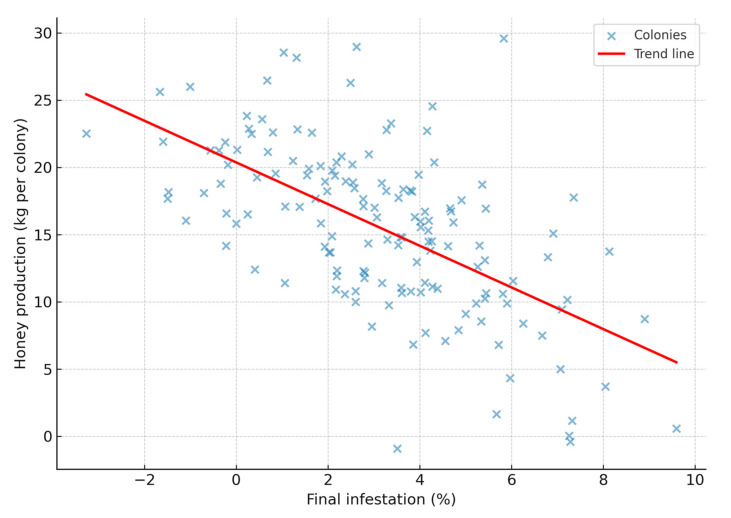
Spearman’s rank correlation between final *Varroa destructor* infestation and honey yield per colony.

These differences in honey yield were closely associated with the level of mite parasitism, as evidenced by a significant negative correlation between final *V*. *destructor* infestation and honey production (Spearman’s ρ = −0.688; 95% CI: −0.764 to −0.594; *p* < 0.0001; n = 150). Colonies with higher mite infestation exhibited markedly reduced honey yields, confirming the strong detrimental impact of *Varroa* on apicultural productivity. These results indicate that acaricide treatments, particularly oxalic acid sublimated, not only reduced mite infestation but also improved honey yield compared with untreated colonies (Figure 2).

### 3.3. Mite Fall

The mixed-effects model revealed significant effects of treatment (F = 102.6, *p* < 0.0001), time (F = 430.0, *p* < 0.0001), and their interaction (F = 40.7, *p* < 0.0001) on mite fall. Colonies treated with oxalic acid sublimated and HappyVarr exhibited the highest mite fall during the first application (18.9 and 16.9 mites, respectively), followed by thymol and oxalic acid/glycerin. Mite fall decreased in subsequent applications, with oxalic acid/glycerin maintaining relatively higher values (6.7 mites at the third application) compared to thymol and HappyVarr. Control colonies consistently showed minimal mite fall (≈3 mites). These results demonstrate that oxalic acid sublimated is the most effective treatment for immediate mite knock-down, while oxalic acid/glycerin provides a more sustained effect over consecutive applications (Table 3).

**Figure 3:**
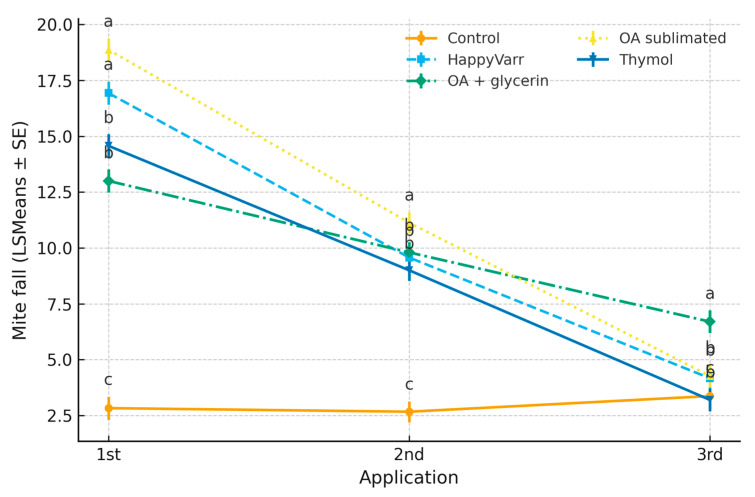
Interaction between acaricide treatment and application number on *Varroa destructor* mite fall (LSMeans ± SE). Different letters above the points indicate statistically significant differences among treatments within each application, according to the Tukey–Kramer multiple comparison test (*p* < 0.05). Treatments sharing the same letter do not differ significantly.

The interaction plot (Figure 3) illustrates that the pattern of mite fall varied markedly across applications depending on the treatment. Oxalic acid sublimated produced the greatest initial mite fall during the first application (18.9 mites), followed by a sharp decline in the second and third applications. Conversely, oxalic acid + glycerin showed a more sustained effect, with moderate values in the first two applications and the highest mite fall in the third application (6.7 mites), outperforming the other acaricides at that stage. HappyVarr and thymol exhibited intermediate efficacy with progressive decreases over time, while the control group remained consistently low across applications. These results confirm a strong treatment × time interaction, indicating that the relative effectiveness of each acaricide changes depending on the application number.

## 4. Discussion

In the present study, all acaricide treatments significantly reduced *Varroa destructor* infestation levels compared to the untreated control, with oxalic acid sublimated and thymol showing the highest efficacy (>75% reduction) according to the Henderson–Tilton formula. These results are consistent with those reported by Adjlane et al. (2016) [30], who also demonstrated substantial reductions in mite infestation following oxalic acid treatments, with final infestation values markedly lower than controls. Similarly, Thurston et al. [31] found that oxalic acid combined with gl ycerin achieved significant decreases in mite levels under field conditions, supporting our observation that this treatment maintained sustained efficacy over multiple applications.

The acaricidal effects of natural compounds have shown considerable variability due to multiple factors. However, first-choice treatments such as oxalic acid and thymol, under practical conditions, highlight the need for continuous research due to variability in their efficacy associated with environmental and genetic factors, as well as the influence of variables such as temperature, humidity, formulation, and application method, which may affect their performance under field conditions [32].

Furthermore, the study of different oxalic acid concentrations by Ahmad et al. [33] confirmed that both dose and seasonality influence final infestation levels, which may explain why the relative efficacy of sublimated vs. glycerin-impregnated oxalic acid differed slightly across applications in our trial.

Adjlane et al. (2020) [34] evaluated the efficacy of thymol (Thymovar^®^) and oxalic acid, demonstrating that thymol achieved the highest acaricidal effectiveness under field conditions. Colonies treated with two half-strips of Thymovar^®^ applied twice at two-week intervals achieved an average efficacy of 90.61%, whereas colonies receiving a single half-strip reached only 64.31% control. In contrast, colonies treated with oxalic acid by the trickling method at concentrations of 45 g/L and 30 g/L achieved 76.35% and 67.52% efficacy, respectively. Overall, thymol exhibited 15–20% higher efficacy than oxalic acid under comparable environmental conditions, confirming its value as a biocompatible, safe, and residue-free acaricide.

Hýbl et al. (2021) [35] reported the use of thyme oil (*Thymus vulgaris*) containing approximately 41% thymol and 16.7% p-cymene. After four hours of exposure, thymol showed a selectivity ratio (SR) of 6.85, one of the highest among the essential oils tested (including peppermint, manuka, oregano, and litsea), indicating high toxicity to mites and low toxicity to honey bees. However, this value decreased over time, reaching 4.56 selectivity ratio (SR) at 72 h, revealing a decline in prolonged efficacy compared with other essential oils such as peppermint and manuka. This decreasing trend aligns with previous findings by Damiani et al. (2009) [36], likely due to thymol’s high volatility and loss of effective concentration over time. Nevertheless, thymol maintained a considerably higher efficacy than the negative control and continues to be regarded as a reference standard for evaluating other essential oils.

Ozuicli et al. (2024) [37] reported that oxalic acid impregnated in glycerin-saturated absorbent towels represented the most effective treatment against *V*. *destructor* under field conditions, achieving over 90% efficacy without adverse effects on honey bees. Additionally, thyme (thymol) and eucalyptus oils also showed high acaricidal activity, with efficacy values close to 80%.

García-Vicente et al. (2025) [38] evaluated mixtures of oxalic acid combined with liquid bacterial metabolite preparations derived from beneficial strains (mainly *Lactobacillus* and *Bifidobacterium*), capable of producing bioactive compounds that modulate the honey bee microbiome and enhance resistance to stress and pathogens. The mixture was applied using the trickling method, directly over adult bees (5 mL per frame space), in three consecutive applications at seven-day intervals during summer and autumn. The combined treatment showed the highest efficacy (~88%), with significant reductions in *V*. *destructor* populations in both adult bees and brood. While postbiotics alone are ineffective, they enhance the acaricidal performance of oxalic acid, offering a promising organic and complementary strategy for integrated pest management (IPM) programs in apiculture.

Glavan et al. (2020) [39] described thymol as a highly effective acaricide against *V*. *destructor*, with dose-dependent effects. Concentrations above 0.05% significantly increased acetylcholinesterase (AChE) and glutathione S-transferase (GST) activities in honey bees, indicating a physiological stress response and activation of detoxification mechanisms. At a 1% concentration, thymol caused approximately 45% mortality, whereas concentrations between 0.05% and 0.5% maintained high acaricidal efficacy without severe toxicity. Thymol acts as a negative allosteric modulator of RDL (resistance to dieldrin) receptors in *V. destructor*, reducing GABAergic conductance and causing paralysis and death of the mite, while in honey bees it produces the opposite effect (positive modulation without inhibition). This mechanism explains the high selective efficacy of thymol as a natural acaricide, achieving up to 95% mite elimination in colonies without compromising bee health [40]. These findings confirm that thymol, when applied at controlled concentrations, constitutes a highly effective and safe natural tool within integrated management strategies for *V. destructor*, although optimization of dosage and application frequency remains essential to prevent potential metabolic disturbances in *Apis mellifera*.

It is also important to consider the seasonal dynamics of mite populations. According to Gamal et al. [10], autumn invasion rates and brood presence strongly affect the outcome of treatments, with higher initial infestations often leading to less dramatic relative reductions. Our findings support this, as colonies with higher baseline infestations still exhibited significant decreases after treatment, but did not always achieve full suppression, reinforcing the need for integrated management strategies. Overall, these results confirm that oxalic acid (particularly sublimated) and thymol remain highly effective tools for *Varroa* management, in line with the international literature, while also emphasizing that the timing of application and baseline infestation levels are critical determinants of treatment success [41,42].

In agreement with Prouty et al. [43], who reported that sublimation of oxalic acid at a dose of 4 g every 5–7 days resulted in substantial reductions in mite infestation, our results with oxalic acid sublimation also demonstrated the highest efficacy among treatments, with very low final infestation percentages. However, the differences observed across our applications indicate that frequency and dosage are key determinants of treatment success. Whereas they used shorter intervals and achieved nearly complete suppression, less frequent applications or lower dosages, as in our case, yielded strong but incomplete reductions. Similarly, Berry et al. [3] found that repeated vaporization of 1 g was effective, although a decline in efficacy was evident in successive applications, particularly when brood reappeared. Our findings also showed a progressive decrease in efficacy from the first to the third application, which may be attributable to the reemergence of brood cells harboring mites inaccessible to treatment, or to residual infestation persisting in the colonies. Although oxalic acid treatments, particularly sublimation, proved to be highly effective in reducing *V. destructor* infestation in our study, the literature emphasizes that potential negative or sublethal effects must also be considered. Tellarani-Prieto et al. [44] demonstrated that very high doses of vaporized oxalic acid (up to 20 g, far exceeding label recommendations) significantly increased worker mortality, although queens and sperm quality were unaffected. In contrast, our treatments employed moderate doses and no increased mortality was observed, reinforcing the importance of respecting application limits. These findings suggest that while adjustments in oxalic acid concentration or frequency might further enhance efficacy, such modifications could also introduce detrimental side effects on colony health. Beyond acute mortality, Sagona et al. [45] demonstrated that oxalic acid can damage midgut tissue and alter immune-related enzyme activity (e.g., glucose oxidase, vitellogenin) under laboratory conditions, suggesting that even when external colony health appears unaffected, sublethal physiological stress may occur. Similarly, Majchark et al. [46] found that exposure to higher concentrations or prolonged contact with oxalic acid can disrupt antioxidant enzyme activity, raising the possibility of oxidative stress within colonies. These findings underscore the need for complementary assessments in field studies, such as immune or enzymatic biomarkers, to fully evaluate the safety profile of acaricidal protocols. Higes et al. [16], reported colonies treated with oxalic acid exhibited significant negative effects on brood development and queen survival when monitored three to four months post-treatment. In our study, short-term colony strength and honey production were not negatively affected, but extended follow-up would be necessary to ensure that repeated or seasonal applications do not compromise brood viability or colony reproductive capacity.

The thymol treatments significantly reduced *V. destructor* infestation, although their efficacy was moderate compared to oxalic acid-based protocols. These findings are consistent with previous reports indicating that thymol can provide substantial control but with variable outcomes depending on formulation, application interval, and environmental conditions. For instance, Floris et al. [13] demonstrated that Apiguard^®^ gel achieved up to 90–95% efficacy under Mediterranean conditions, whereas Api Life VAR^®^ wafers were somewhat less effective, and colony-level variability was evident. Our results align with this variability, suggesting that differences in brood presence, colony strength, and local climate (temperature and humidity) may have limited the overall reduction in mite levels. Recent advances in formulation technology, such as thymol nanoemulsions, have also shown promise; Gamal-Eldin et al. [10] reported that nanoemulsified thymol exhibited high acaricidal activity while maintaining colony safety. Moreover, Gregorc et al. [11] confirmed that thymol generally exerts low toxicity on adult bees when applied appropriately, supporting its suitability as a “soft acaricide.” Frey et al. and Chinkangsadarn et al. [47,48] demonstrated that when applied at regular intervals, this treatment can achieve very high reductions in infestation, approaching 98–100% under favorable conditions. In contrast, our results revealed a significant but lower reduction, which may be explained by differences in environmental factors (e.g., temperature, humidity), baseline infestation density, or the presence of brood that provides refuge for mites. Taken together, these results indicate that while thymol was not the most effective treatment in our trial, it remains a valuable option in integrated pest management programs, especially when formulated or applied under conditions that optimize its volatility and persistence.

The integrated pest management (IPM) is not limited to product application but rather integrates monitoring, knowledge of brood cycles, and treatment timing [49]. Poorly synchronized treatments can reduce honey production despite improving *Varroa* control [50]. In contrast, aligning treatments with periods of reduced brood enhances contact efficacy and reduces the need for higher dosages [51]. Our results, where the relative efficacy of each treatment varied across applications, are consistent with the notion that brood dynamics and timing of intervention are key determinants. O’Connell et al. [52] further indicated that under appropriate conditions, organic treatments (oxalic acid, formic acid, thymol) can achieve efficacies comparable to synthetic acaricides, with the added benefit of avoiding undesirable residues [2]. From a production standpoint, this means that beekeepers can maintain or even improve honey yields if treatment windows are respected, modes of action are rotated, and interventions are applied based on infestation thresholds [53,54,55]. The present study, showing significant reductions in mite infestation accompanied by increases in honey production, provides practical evidence supporting this benefit.

Ultimately, the integration of organic acaricides within an IPM framework underscores the importance of considering not only treatment efficacy but also the complex biological interactions that shape colony performance [56]. *V. destructor* parasitism simultaneously affects honey bee physiology, viral transmission dynamics, and colony productivity, meaning that treatment outcomes cannot be evaluated in isolation [5,9]. Our findings demonstrate that effective suppression of mite populations through oxalic acid and thymol treatments translates into measurable improvements in honey yields, reinforcing the close linkage between parasite pressure and productive performance. These results highlight that sustainable apiculture requires a holistic perspective, where the health of the bee, the biology of the mite, and the timing and type of intervention are integrated into management decisions. By aligning acaricide efficacy with colony productivity, this study provides evidence that IPM strategies based on natural treatments can promote both effective *Varroa* control and long-term beekeeping sustainability [57,58].

## 5. Conclusions

This study demonstrates that organic and commercial acaricides can effectively reduce *Varroa destructor* infestation while simultaneously improving honey production under the semi-arid subtropical conditions of Northeastern Mexico. Among the treatments evaluated, sublimated oxalic acid showed the highest efficacy, providing rapid mite suppression and the greatest honey yields, whereas thymol and HappyVarr^®^ also achieved significant reductions with favorable impacts on productivity. A strong negative correlation between final infestation levels and honey production confirmed the detrimental impact of *Varroa* on colony performance and the benefits of effective control. These findings highlight the importance of incorporating organic treatments within integrated pest management (IPM) programs, where timing of application, brood dynamics, and rotation of active ingredients are essential to maximize both colony health and productivity. By aligning mite control with honey yield outcomes, this study provides practical evidence that IPM strategies based on natural treatments offer a sustainable pathway for *Varroa* management in apiculture.

## Figures and Tables

**Table 1:** Final infestation percentages of *Varroa destructor* in *Apis mellifera* colonies treated with different acaricides (Kruskal–Wallis test).

Treatment	Colonies	Median (IQR)
Control	30	8.40 (7.69–8.82) ^a^
HappyVarr	30	1.84 (1.49–2.53) ^b^
Oxalic acid + glycerin	30	2.30 (1.89–2.93) ^b^
Oxalic acid sublimated	30	1.97 (1.57–2.45) ^b^
Thymol	30	2.38 (1.80–2.73) ^b^

IQR = interquartile range. Different superscript letters indicate significant differences among treatments according to the Steel–Dwass test (*p* < 0.05).

**Table 2:** Effectiveness of organic treatments in reducing *Varroa destructor* infestation (Henderson and Tilton equation).

Treatment	Initial Infestation (%)	Final Infestation (%)	Efficacy (%)
Control	6.9	8.5	-
HappyVarr	6.9	2.3	72.9
Oxalic acid + glycerin	6.7	2.3	72.1
Oxalic acid sublimated	7.3	2.0	77.8
Thymol	7.2	2.0	77.5

**Table 3:** Mite fall in *Apis mellifera* colonies treated with different acaricides across three consecutive applications.

Treatment	1st Application (LSMean ± SE)	2nd Application (LSMean ± SE)	3rd Application (LSMean ± SE)
Control	2.83 ± 0.52 ^c^	2.67 ± 0.47 ^c^	3.37 ± 0.51 ^c^
HappyVarr	16.93 ± 0.52 ^a^	9.57 ± 0.47 ^b^	4.17 ± 0.51 ^b^
Oxalic acid + glycerin	13.00 ± 0.52 ^b^	9.80 ± 0.47 ^b^	6.70 ± 0.51 ^a^
Oxalic acid sublimated	18.87 ± 0.52 ^a^	11.13 ± 0.47 ^a^	4.27 ± 0.51 ^b^
Thymol	14.57 ± 0.52 ^b^	14.57 ± 0.52 ^b^	14.57 ± 0.52 ^b^

Mixed model analysis: significant effects of treatment (*p* < 0.0001), time (*p* < 0.0001), and treatment × time interaction (*p* < 0.0001). LSMean = Least-Squares Mean. SE = Standard error. Different superscript letters within each column indicate significant differences among treatments (Tukey’s HSD, *p* < 0.05).

## Data Availability

The original contributions presented in this study are included in the article material. Further inquiries can be directed to the corresponding author.

## Abbreviations

The following abbreviations are used in this manuscript:

T1	Treatment 1
T2	Treatment 2
T3	Treatment 3
T4	Treatment 4
T5	Untreated control
DWV	Deformed wing virus
ABPV	Acute bee paralysis virus
IAPV	Israeli acute paralysis virus
IPM	Integrated pest management
N	North
W	West
°C	Degrees Celsius
mm	millimeter
cm	centimeter
L	Liter
OA	Oxalic acid
g	gram
V	Volts
min	minutes
s	seconds
mL	milliliter
cm^3^	cubic centimeters
Tb	initial infestation in the treatment group
Ta	final infestation in the treatment group
C*b*	initial infestation in the control group
Ca	final infestation in the control group
SAS	Statistical Analysis System
χ^2^	Chi square
df	degree of freedom
